# Treating Hyperexcitability in Human Cerebral Organoids Resulting from Oxygen-Glucose Deprivation

**DOI:** 10.3390/cells12151949

**Published:** 2023-07-27

**Authors:** Alexandra C. Santos, George Nader, Dana El Soufi El Sabbagh, Karolina Urban, Liliana Attisano, Peter L. Carlen

**Affiliations:** 1 Krembil Research Institute, University Health Network, Toronto, ON M5S 0T8, Canadapeter.carlen@uhnresearch.ca (P.L.C.); 2 Institute of Biomedical Engineering, University of Toronto, Toronto, ON M5S 1A1, Canada; 3 Department of Pharmacology and Toxicology, University of Toronto, Toronto, ON M5S 1A1, Canada; 4 Avicanna Inc., Toronto, ON M5G 1V2, Canada; 5 Department of Biochemistry, University of Toronto, Toronto, ON M5S 1A1, Canada; 6 Donnelly Centre, University of Toronto, Toronto, ON M5S 1A1, Canada

**Keywords:** organoids, electrophysiology, epilepsy, brain, GABA

## Abstract

Human cerebral organoids resemble the 3D complexity of the human brain and have the potential to augment current drug development pipelines for neurological disease. Epilepsy is a complex neurological condition characterized by recurrent seizures. A third of people with epilepsy do not respond to currently available pharmaceutical drugs, and there is not one drug that treats all subtypes; thus, better models of epilepsy are needed for drug development. Cerebral organoids may be used to address this unmet need. In the present work, human cerebral organoids are used along with electrophysiological methods to explore oxygen-glucose deprivation as a hyperexcitability agent. This activity is investigated in its response to current antiseizure drugs. Furthermore, the mechanism of action of the drug candidates is probed with qPCR and immunofluorescence. The findings demonstrate OGD-induced hyperexcitable changes in the cerebral organoid tissue, which is treated with cannabidiol and bumetanide. There is evidence for NKCC1 and KCC2 gene expression, as well as other genes and proteins involved in the complex development of GABAergic signaling. This study supports the use of organoids as a platform for modelling cerebral cortical hyperexcitability that could be extended to modelling epilepsy and used for drug discovery.

## 1. Introduction

Advances in stem cell technology have made it possible to create cerebral organoids from human embryonic stem cells (ESCs) and induced pluripotent stem cells (iPSCs). Cerebral organoids are 3D self-organizing neuronal tissue constructs that resemble several features of the developing human brain [1,2]. Organoids permit the study of human tissue, which has historically been limited by the low availability of donated brain tissue. There is no reliance on a specific patient per se; therefore, cerebral organoids can be made without direct patient involvement. Furthermore, cerebral organoids can be grown in bulk to allow for high-throughput analyses, such as drug action or toxicity screening. 

Cerebral organoid tissue briefly resembles the fetal cortex in multiple domains [3,4]. Due to this characterization, the tissue can be useful for studying neurodevelopment and pathology of the neonatal brain. The neonatal period has the highest incidence of seizures, often due to hypoxic ischemic encephalopathy (HIE) [5,6] caused by oxygen-glucose deprivation (OGD) [7]. These types of seizures are often drug-resistant and accompanied by poor neurological outcomes [5,6,8,9]. The present study will use OGD to induce hyperexcitability in the cerebral organoids, then further evaluate the response to drugs that are used in other OGD models.

The immature brain is disproportionately more susceptible to hyperexcitable insults [5,10], in particular due to the altering patterns of GABAergic inhibition. Briefly, GABA begins as a neuronal depolarizing agent during early brain growth, then transitions to a hyperpolarizing agent during postnatal development and continues to dominate as a major neuronal inhibitory molecule throughout development [10]. Due to this central importance in brain excitability, it would be beneficial for cerebral organoids to model this aspect of neurodevelopment, as it can further support the tissue’s resemblance to unique human physiology, and to investigate diseases that involve hyperexcitability, such as epilepsy.

In the present study, OGD was used to induce a hyperexcitable state in human cerebral organoids during an acute electrophysiological experiment. This state was then modified with various compounds that are known to have an effect on OGD, including bumetanide and cannabidiol. Bumetanide is a drug that blocks NKCC1, a chloride transporter expressed in neurons. During early development, there is more NKCC1 than KCC2 (another chloride transporter), which results in intracellular chloride accumulation, causing GABA to have depolarizing actions [10]. Blockade with bumetanide has antiseizure effects in neonatal OGD and hypoxia seizure models [11,12,13]. Therefore, it was investigated whether bumetanide can elicit similar effects in preventing OGD-induced changes in 4- and 7-month organoids.

Cannabidiol (CBD) is one of the active components in cannabis and has both antiseizure [14] and antioxidant [15,16,17] properties. Furthermore, CBD is approved to treat Dravet Syndrome, which is a childhood epileptic encephalopathy [18]. It has been demonstrated that cannabidiol has neuroprotective properties in various models of hypoxia–ischemia [19,20,21]; therefore, CBD was used to investigate if it could prevent the power spectral hyperexcitability previously observed to be induced by OGD treatment in cerebral organoid tissue.

This study demonstrates that OGD can be used to induce hyperexcitability in organoids, which can be treated with bumetanide and cannabidiol. There is evidence of the expression of different genes which may contribute to OGD’s effects. Overall, cerebral organoids can be used to model brain excitability, such as in epilepsy, and to further characterize key developmental milestones, such as the GABA switch, warranting the use of human cerebral organoids for modelling human disease and evaluation of drug candidates.

## 2. Materials and Methods

### 2.1. Cerebral Organoid Generation and Maintenance

Cerebral organoids (COs) were generated from H9 and H1 human embryonic stem cell lines in the Applied Organoid Core (ApOC), University of Toronto as previously described [22]. Organoids were grown and maintained in 6-well plates (maximum 4 organoids per well), immersed in 3 mL of growth media, and kept in a 37 °C incubator with 5% CO_2_ and room oxygen (21%). For media changes, 1.5 mL of old media was discarded and replaced with 1.5 mL fresh media every 3–4 days. 

### 2.2. Immunofluorescence and Microscopy

COs were washed three times, 10 min each with OBS in 1.5 mL Eppendorf tubes and then fixed with 4% PFA in 0.1% Tween 20 in PBS (PBST) overnight at 4 °C on a rocker. COs were embedded in 15% sucrose for 3 h, followed by 30% sucrose overnight at 4 °C. COs were placed on cryomolds, and OCT was added while placed on dry ice to flash freeze. Cryomolds were transferred to −80 °C until cryosectioning.

The samples were cryosectioned using the Leica CM3050S cryostat and 20 µm a slice and mounted on SuperFrost Plus microscope slides (22-037-246; Thermo Fisher Scientific, Toronto, ON, Canada).

Samples were washed three times, 5 min each in PBST at room temperature, and blocked and permeabilized using 0.5% Triton-X 100 and 2% BSA in PBST for one hour at room temperature in a humid chamber. Samples were then stained with primary antibodies (Supplementary Table S1) diluted using PBST, and incubated overnight at 4 °C in a humid chamber. Samples were again washed three times in PBST and secondary antibodies (1:750), diluted in PBST were added. Incubation was for 1 h room temperature in a humid chamber. The slides were washed three times in PBST and mounted using DAPI Fluoromount-G mounting media (0–100 20, Southern Biotech, Birmingham, AL, USA) with glass coverslips and left to dry in the dark overnight. Samples were imaged using a Zeiss LSM 880 Super resolution confocal microscope from University of Toronto Microscope Imaging Laboratory. Images were processed using FIJI (ImageJ) software (version 2.7.0). Samples were stained with secondary antibody only to confirm that no nonspecific binding occurred (Supplementary Figure S1).

### 2.3. Acute Slice Electrophysiology

#### 2.3.1. Acute Slice Preparation

For all electrophysiological recordings, slices were cut the day of experimentation and discarded immediately after each recording. Whole cerebral organoids were embedded in 3% low-gelling temperature agarose (A9045, Sigma-Aldrich, Milwaukee, WI, USA), and double distilled water that was 37 °C, then cooled on ice for 5–10 min. Next, the embedded cerebral organoid was glued to a mounting chuck, immersed in chilled 4 °C sucrose dissection solution [in mM: 87 NaCl, 25 NaHCO_3_, 25 glucose, 2.5 KCl, 7 MgCl_2_, 1.25 NaHPO_4_, 0.5 CaCl_2_, 75 sucrose], and sliced into 400 µm thick slices with a Leica VT1200 vibratome with a 0.6 mm/s advancement speed and 1 mm horizontal displacement. Slices were transferred to an incubation chamber at 37 °C filled with artificial cerebral spinal fluid (ACSF) [in mM: 125 NaCl, 25 NaHCO_3_, 10 glucose, 2.5 KCl, 1.5 MgCl_2_, 2.5 CaCl_2_] perfused with carbogen (95% O_2_, 5% CO_2_). Slices were recovered for a minimum of 15 min, then removed from the 37 °C water bath and left at room temperature (21 °C) in the perfusion chamber for a minimum of 30 min before electrophysiological recordings.

#### 2.3.2. Data Acquisition

Slices were placed in a recording chamber perfused with carbogenated ACSF heated to 37 °C. Local field potential (LFP) recordings were performed using electrodes pulled from borosilicate capillary glass, subsequently filled with ACSF. Electrodes were positioned 150 µm deep from the outer rim of each slice, on opposite sides of the tissue. Recordings were 5 min of baseline (regular ACSF), 15 min of OGD [in mM: 125 NaCl, 25 NaHCO_3_, 2.5 KCl, 1.5 MgCl_2_, 2.5 CaCl_2_, 10 sucrose, no carbogenation], followed by 15 min for washout (regular ACSF). The pH of the OGD was balanced to 7.3–7.4 prior to experiments. Signals were acquired with Digidata 1322A digitizer (Axon Instruments) and Multiclamp 700B amplifier (Molecular Devices) using PClamp software (version 10.2) at a sampling rate of 25,000 Hz.

#### 2.3.3. Power Spectral Density Analysis and Statistics

The PSD is a commonly used analysis of electroencephalography (EEG) signals [23]. The PSD breaks down a signal into its various frequency components, including delta (1–4 Hz), theta (4–8 Hz), alpha (8–13 Hz), beta (13–30 Hz), and gamma (30–80 Hz), and quantifies the distribution of power each of those frequencies holds [23,24]. The higher the PSD value, which may be quantified as area under the curve, the more activity in the EEG [23]. In humans, the use of EEG is a standard practice in the diagnosis and evaluation of various epilepsies [24]. Thus, the PSD can be a valuable measure of various brain states during seizures.

The raw signals were processed for power spectrum analysis in MATLAB. Raw signals were not visualized in the present study; however, a detailed figure outlining the raw traces and corresponding power spectrum can be found in Figure 2 from [25]. LFP recordings were filtered using a lowpass filter at half the sample rate, and subsequently down sampled by a factor of 2, split over 3 cycles. A 60-notch filter (with 5 harmonics) was then applied to remove noise. The traces for each condition were z-scored by the mean of the baseline, for each slice. The traces were then split into 10 s sections (with 50% overlap), a Hann window was applied to each sectioned signal, and the fast Fourier transform (FFT), and power spectral density (PSD) were computed per slice. The area under the curve (AUC) of the PSD plots was calculated by taking the sum of binned frequencies over specific frequency ranges.

The area under the curve (AUC) calculated from the power spectrum was used to compare conditions quantitatively in the cerebral organoids. Firstly, outliers in the average AUC for each channel were removed using the quartile method. Both channels from the same slice were then averaged to give each slice one AUC value for each frequency. The frequencies of interest were delta (1–3.9 Hz), theta (4–7.9 Hz), alpha (8–12.9 Hz), beta (13–29.9 Hz), gamma (30–79.9 Hz), high-frequency oscillations (80–119.9 Hz), and very-high-frequency oscillations (120–999.9 Hz). While high-frequency oscillations can also be classified as ripples (80–200 Hz) and fast ripples (250–500 Hz), there was not an indication from the raw data to differentiate between these frequencies; therefore, they were grouped into high- or very-high-frequency oscillations. A *t*-test was used to compare any two mean AUC values, and *p*-values were Bonferroni corrected. Furthermore, average percent change in AUC from baseline to selected condition (convulsant, or washout) was calculated for each slice as ((final value-initial value)/initial value) × 100, and used to compare across groups (e.g., percent change from baseline to OGD at 4 months or 7 months).

### 2.4. RT-qPCR

Both 4- and 7-month H9 cerebral organoids [22] were flash-frozen in dry ice once at the desired age and stored in a −80 °C freezer until further processing. The organoids were placed in sterilized 1.5 mL Eppendorf tubes, and the media were aspired. TRIzol reagent (Sigma, 15596026) was used for RNA extraction, followed by washes in chloroform, isopropanol, and 75% ethanol, resuspension in RNase-free water, and finally, acrylamide (Invitrogen, AM9520). RNA concentration and purity were measured using a nanodrop. Subsequent cDNA synthesis was conducted using 2 µg of RNA with Superscript III (Invitrogen, 18080051) according to the manufacturer’s protocol. Template cDNA was diluted to 250 ng per reaction with yellow dye (Thermo Scientific, R1381) and distilled water. The 10 µL reaction volume contained the following: 2 µL of the template cDNA, 1 µL of forward and reverse primers at 10 µM, and 7 µL of 2X SYBR (Applied Biosystems, 4209155). The forward and reverse primer sequences used for each gene of interest can be found in the Supplementary Table S1.

Four organoid samples were used for each age, and each gene was run in triplicate. Reagents were pipetted into a 384-well PCR plate (Applied Biosystems, 4309849) and sealed with film (Applied Biosystems, 4311971). Plates were briefly centrifuged prior to the reaction, which was carried out using QuantStudio5 machine (272530542) using the following reaction protocol: hold phase (95 °C for 5 min), PCR phase (95 °C for 15 s, 58 °C for 1 min, 40 cycles), and melting phase (95 °C for 15 s, 58 °C for 1 min, 95 °C for 15 s).

### 2.5. qPCR Data Analysis and Statistics

Prior to data analysis, the melting curves of each replicate were analyzed and compared to the values of the no-template control (NTC). Additionally, Ct values above 36 were removed [26]. Replicates were averaged, and the DCt and DDCt for each gene of interest was calculated using the following formula:

DCt = Ct (gene of interest) − Ct (housekeeping gene).

DDCt = DCt _7months_ − DCt _4months_

Additionally, the relative expression of organoids between 7 and 4 months was calculated according to the following formula:

Relative Expression = 2^−DDCt^

This was plotted in R to display the relative expression of various genes. The mean DCt values from each gene were compared between ages with a Student’s *t*-test.

## 3. Results

### 3.1. Immunofluorescence Demonstrates Presence of Neurons

**Figure 1:**
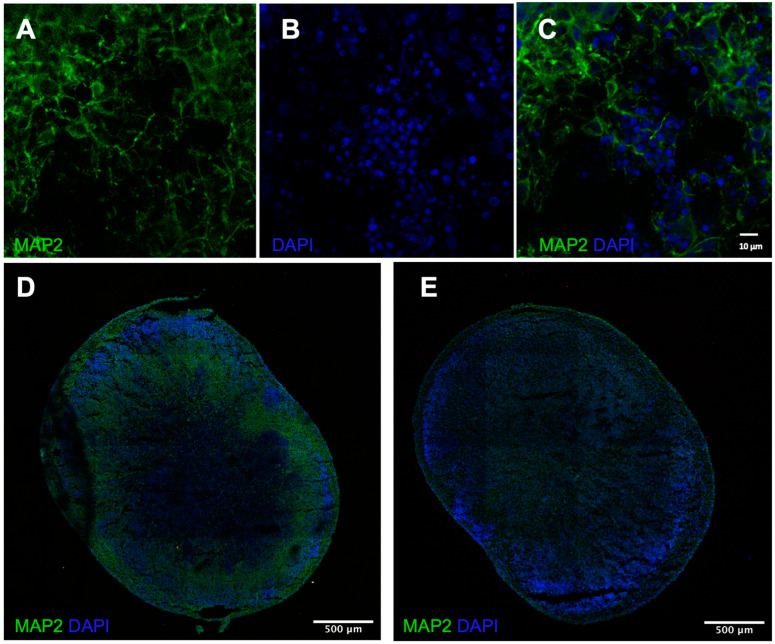
Immunofluorescence and microscopy results. (**A**,**B**) Staining of MAP2 and DAPI individually in 7-month H9 cerebral organoids at 63× magnification oil immersion Zeiss LSM 880 Super resolution confocal microscope from University of Toronto Microscope Imaging Laboratory. (**C**) Colocalized stain on 63× and (**D**) 10× magnification, Zeiss LSM 880 Super resolution confocal microscope from University of Toronto Microscope Imaging Laboratory. (**E**) Colocalized staining of GFAP and DAPI in 7-month H9 cerebral organoids at 10× magnification oil immersion Zeiss LSM 880 Super resolution confocal microscope from University of Toronto Microscope Imaging Laboratory.

To characterize neurons evident in the cerebral organoid tissue, immunofluorescence was conducted with DAPI (nucleus), MAP2 (neurons), and GFAP (astrocytes). The signal for DAPI, MAP2, and GFAP was robust, indicating neurons and astrocytes present in the tissue (Figure 1), and providing support for the electrophysiological experiments. Future work with 4-month tissue is necessary to compare the temporal protein expression and relate it back to the qPCR and electrophysiological results. More in-depth characterization of the H9 organoids can be found in Figure 2 of Sivitilli et al. [22].

### 3.2. OGD Is an Effective Hyperexcitable Agent at 4 and 7 Months

**Figure 2:**
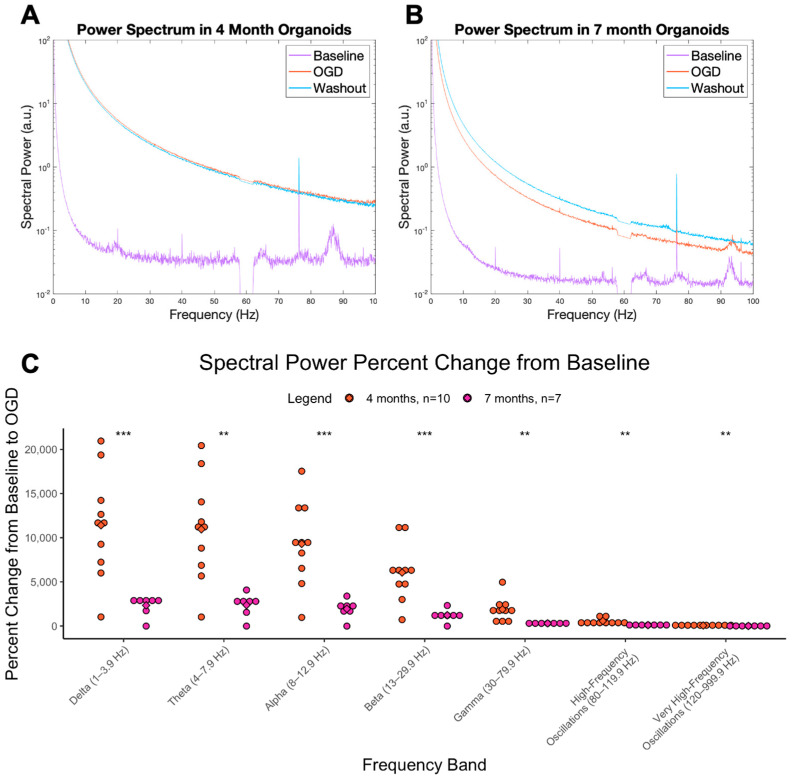
OGD power spectrum and change from baseline. Power spectrum of cerebral organoids that underwent baseline, OGD, and washout treatments at (**A**) 4 months and (**B**) 7 months. The y axis is spectral power in arbitrary units and the x axis is the frequency. (**C**) Percent change in spectral power as AUC from baseline condition to OGD condition, at 4 (orange) and 7 months (pink). The y axis represents the percent change (increase) in the AUC from baseline to OGD. The x axis represents the 7 different brain wave frequencies. *** = *p* < 0.001, ** = *p* < 0.01.

OGD induces a global increase in the power spectrum compared to baseline in cerebral organoids aged 4 and 7 months (Figure 2A,B). This effect is the same as that reported in our previous work [25,27]. The percent change from baseline to OGD was significant across most frequencies at 4 and 7 months (Figure 2C); however, it was more significant at 4 months (delta-beta, *p* < 0.001, gamma-vHFOs, *p* < 0.01), suggesting OGD is effective at inducing a hyperexcitable state in immature tissue. A sample trace demonstrating hyperexcitable; spiking events can be found in Figure S2. These results are the basis for using OGD in cerebral organoids as a model of neonatal seizure-related hyperactivity; thus, the next step was to investigate if the OGD-induced hyperexcitability could be prevented by various drugs.

### 3.3. Bumetanide Significantly Reduces OGD-Induced Excitability

**Figure 3:**
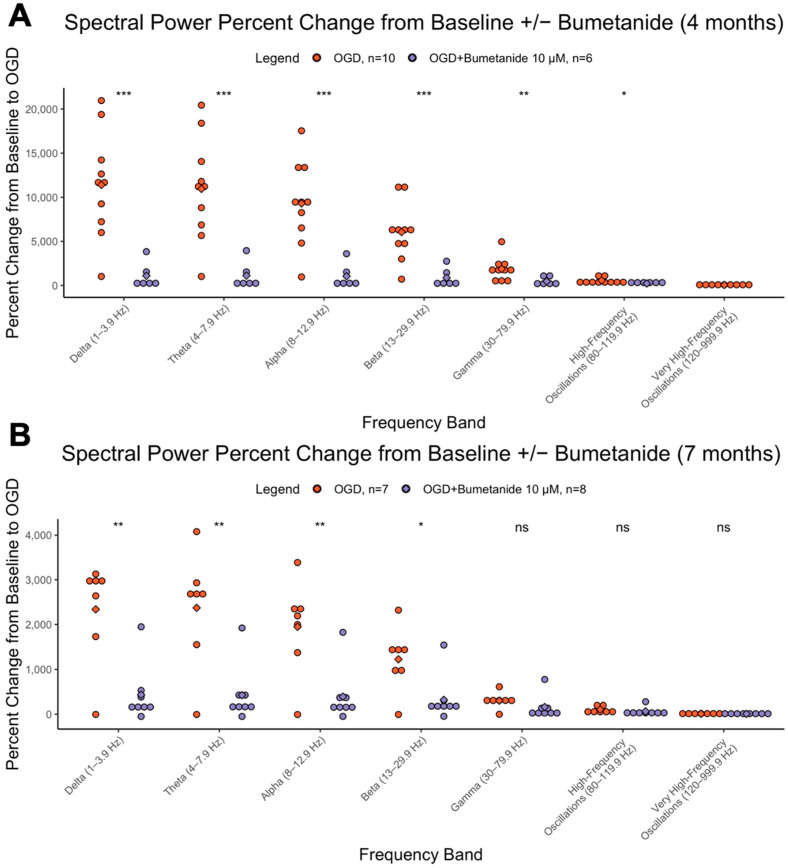
Effects of bumetanide on spectral power. Percent change in spectral power from baseline to OGD in cerebral organoids that underwent bumetanide or no pretreatment at (**A**) 4 months and (**B**) 7 months. The y axis represents the percent change (increase) in the AUC from baseline to OGD. The x axis represents the 7 different brain wave frequencies. Orange is OGD alone, and purple is OGD after bumetanide exposure. *** = *p* < 0.001, ** = *p*< 0.01, * = *p* < 0.05, ns = not significant.

After 5 min of baseline recording, slices underwent 15 min bumetanide 10 µM, then 15 min OGD + bumetanide 10 µM, then 15 min washout. Bumetanide reduced the large increases in spectral power observed typically during OGD. This effect, as depicted by percent change from baseline, was significant at most frequencies in 4-month organoids (delta-beta, *p* < 0.001, gamma-HFOs, *p* < 0.01, vHFOs, *p* = ns) and was significant for delta through beta frequencies at 7 months (delta-alpha *p* < 0.01, beta *p* < 0.05) (Figure 3A,B). This effect is quantified comparing the OGD state in bumetanide-treated and nontreated organoids at both ages. Interestingly, bumetanide’s suppression of the power spectrum was found to be higher in 4-month organoids relative to 7-month ones, when comparing the percent change in spectral power from baseline to OGD + bumetanide (Table 1).

### 3.4. Cannabidiol Reduces OGD-Induced Hyperexcitability at 4 Months

**Figure 4:**
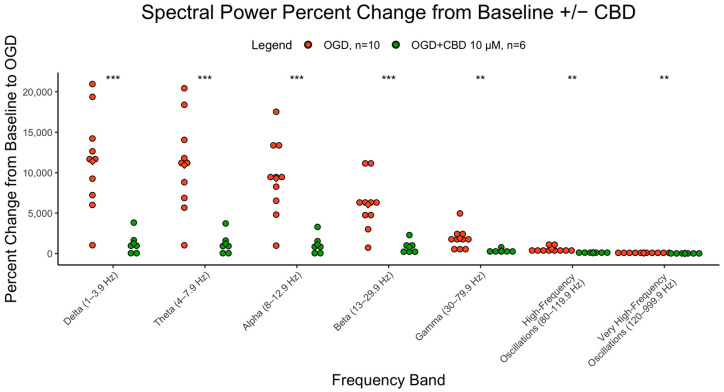
Effects of CBD on spectral power. Percent change in spectral power from baseline to OGD in cerebral organoids that underwent CBD or no pretreatment at 4 months. The y axis represents the percent change (increase) of the AUC from baseline to OGD. The x axis represents the 7 different brain wave frequencies. Orange is OGD alone, and green is OGD after CBD exposure. *** = *p* < 0.001, ** = *p*< 0.01.

CBD at 10 µM was tested in 4-month organoids that underwent exposure to OGD. The percent change from baseline to OGD was significantly lower in CBD-treated slices compared to normal slices that underwent OGD exposure (delta-beta, *p* < 0.001, gamma-vHFOs, *p* < 0.01). Figure 4 illustrates the percent change from baseline for each frequency in CBD-treated and untreated slices undergoing OGD exposure at 4 months.

### 3.5. Transcriptional Profile at 4 and 7 Months through RT-qPCR

**Figure 5:**
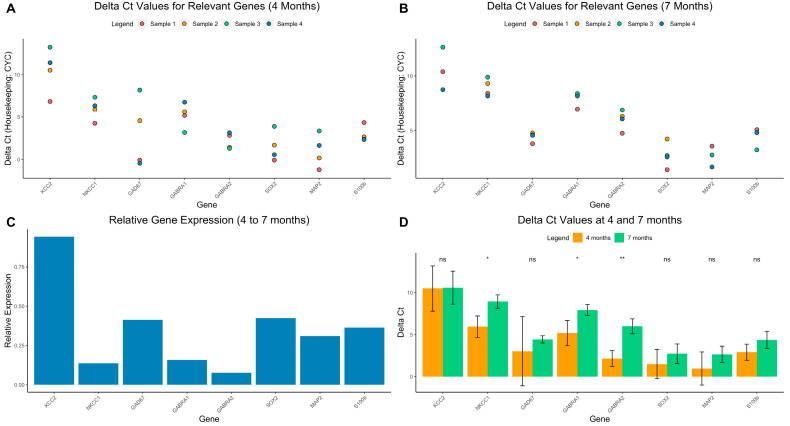
Main qPCR results. ΔCt values from qPCR experiments compared to housekeeping gene CYC at 4 (**A**) and 7 months (**B**). Gene name is on the x-axis and ΔCt is on the y axis. (**C**) Relative expression from 4 to 7 months, calculated by with the formula 2^−DDCt^. (**D**) Mean and standard deviation of ΔCt values compared between ages. ** = *p*< 0.01, * = *p* < 0.05, ns = not significant.

Knowing that bumetanide is a selective NKCC1 inhibitor, we proceeded to investigate if the latter was expressed in cerebral organoids, as well as GABA-related genes. Our qPCR results provided evidence of the presence of GABA-related genes in cerebral organoids at 4 and 7 months, including: GABAA-R (gamma aminobutyric acid receptor-alpha subunits), NKCC1 and KCC2 (chloride transporters), GAD67 (glutamate decarboxylase) (Figure 5A,B). Furthermore, the organoids also demonstrated multiple neuronal markers, such as SOX2 (neuroprogenitor marker), MAP2 (mature neuron marker), and S100b (astrocyte marker). The fold change from 4 to 7 months is visualized in Figure 5C.

### 3.6. GABAergic Profile at 4 and 7 Months

Aiming to explain the anticonvulsant effects of bumetanide, we analyzed the GABAergic profile of the organoids using qPCR. At both 4 and 7 months, NKCC1 expression was higher than KCC2 (DCt values: 4 months: NKCC1: 5.83, KCC2: 10.51, *p* = ns, 7 months: NKCC1: 8.95, KCC2: 10.59, *p*= ns) Interestingly, the expression of NKCC1 decreased at 7 months compared to 4 months (DCt values: 7 months: mean = 8.95, 4 months: mean = 5.83, *p* = 0.02), while KCC2 expression remained relatively constant (DCt values: 7 months: mean = 10.59, 4 months: mean = 10.51, *p* = ns) (Figure 5D). Additionally, GABARA1 and 2 were significantly decreased at 7 months compared to 4 months (Figure 5D).

## 4. Discussion

In summary, cerebral organoids respond to OGD as a hyperexcitability (seizure-like) stimulus, producing significant power changes, which is consistent with previous work completed in the lab [25,27]. Bumetanide and CBD are both effective at preventing the OGD-induced changes, which highlights these compounds’ efficacy in hypoxic–ischemic-induced hyperexcitability and their potential use for treating neonatal hypoxic–ischemic-induced seizures. Furthermore, the mechanism of OGD likely involves a complex interplay of GABAergic signaling, brain development, and intracellular chloride accumulation, as demonstrated by the presence of NKCC1 and KCC2 transporters and GABA-related genes. Further work is required to better elucidate the mechanisms, as well as antiseizure drug responses to develop organoids as a drug testing platform.

While the present study demonstrated a hyperexcitable response to OGD that aligns with features of neonatal hypoxic–ischemic-induced seizures, there is still much characterization before this can be considered a robust model of epilepsy. Epileptiform discharges in vitro are often characterized on appearance, mainly recurrent spiking events that are clear deviations from baseline activity, lasting on the order of a few to many seconds, depending on if it is interictal or ictal activity [28]. While consistent spiking events were not evident in the present study, the power spectrum remains a useful electrophysiological measure of hyperexcitability, which can be extended to epilepsy. Herein, we demonstrate up to a remarkable 10,000% increase in low-frequency spectral power with OGD. For example, seizures have shown increased power in the slower wave spectrum, such as theta [24], and increased delta power has been associated with interictal epileptiform discharges in patients with pharmacoresistant temporal lobe epilepsy [29]. In a rodent model of hypoxia and hypoxic–ischemic-induced seizures, both insults produced an increased in total power (0.5–20 Hz) compared to controls [30]. Overall, the PSD can be a valuable quantification of the overall network activity in live tissue, and thus can aid in classifying hyperexcitable brain states, which could be related to epilepsy.

An important component of disease modelling is demonstrating drug responses similar to in vivo responses. This was the basis for the bumetanide experiments. The NKCC1 blocker that is antiseizure in OGD lab models prevented the hyperexcitable change that OGD typically produced, and this was more pronounced at 4 months. This finding is in line with our qPCR characterization results, as there was greater NKCC1 expression at 4 months, which explains the stronger bumetanide suppression at that age relative to 7 months. This finding parallels other lab models of neonatal seizures, where high NKCC1 is found in early development, and likely contributes to the hyperexcitability [11]. The GABA switch is characterized by NKCC1-dominant switches to KCC2-dominant. The present study demonstrated NKCC1 expression decreasing over time; however, KCC2 was relatively constant, suggesting there are many factors that influence the GABA switch, which may not be present in cerebral organoids. Additionally, immunofluorescence indicated the presence of neuronal cells, which demonstrates that neurons are likely contributing to both the electrophysiological and gene expression results. Overall, cerebral organoid development and function still remains to be characterized in detail, especially in systems that show precise and interactive dynamics during early development.

The present work also demonstrated CBD’s ability to prevent the OGD-induced power increases, validating its known therapeutic properties and parallelling cerebral organoid results with what is known in existing lab models. A study that investigated CBD in newborn piglets exposed to hypoxic–ischemic conditions found that amplitude-integrated EEG returned to 86% of the baseline activity, with neurobehavioural benefits after CBD treatment, when compared to the vehicle [31]. Neonatal hypoxia–ischemia can lead to seizures and epilepsy [32], which may become resistant to the antiseizure medications currently available. The immature brain is different in its susceptibility to developing epilepsy; therefore, it would be warranted to investigate treatments for neonatal seizures in a tissue that models the immature human brain. CBD demonstrates efficacy in drug-resistant epilepsy [18]. Thus, investigating whether CBD has efficacy in treating neonatal seizures may be a novel therapeutic avenue.

Overall, these findings suggest that cerebral organoids may have underlying developmental mechanisms that require further study, as well as the tissue’s utility in demonstrating hypoxic–ischemic-related hyperexcitability and drug responses.

## 5. Limitations

There are several limitations to the present work. It is evident that applying acute slice electrophysiology methods to cerebral organoid tissue may not be optimal and may be damaging the tissue. While these methods are commonly employed in mammal brain tissue, the cerebral organoids do not have robust structure and integrity yet, and therefore damage could be occurring that is not directly observable. To overcome this limitation, the use of chronically cultured tissue on MEA plates is warranted. This will allow for minimal disturbance of the tissue’s growth and structure, and permits a long-term evaluation of the cerebral organoids’ electrophysiological function. Additionally, the cell damage that can result from OGD was not assessed in this study. This is necessary for future work to determine the mechanisms of OGD, and if it is accurately modelling the conditions and pathology seen in human tissue exposed to OGD. Future work is also needed to investigate other antiseizure drugs, such as phenobarbital, which is the first line of treatment for neonatal epilepsy [33].

While a small subset of genes was investigated with qPCR, it would be beneficial to further probe the tissue for the transcriptional profile and include more timepoints. Most results in the present study included both 4 and 7 months; however, the CBD results were only at 4 months, and immunofluorescence was only conducted at 7 months. Inclusions of the respective timepoints is warranted to better understand CBD’s effects. Additionally, this study focused on cerebral organoids that did not receive directed differentiation such as with SMAD inhibition to create GABAergic cell types; therefore, the use of ganglionic eminence-derived organoids [34] would be useful to compare GABA cell development in different tissue types.

Another limitation of using cerebral organoids is the lack of vascularization. As organoids form 3D connections, the core lacks perfusion of growth media and vital gases for survival, thus becoming necrotic [35]. The present study attempted to overcome this limitation by recording near the outer edge of the tissue; however, better methods of sustaining optimal tissue health are necessary, such as a vascular scaffold [36,37] or co-culturing organoids with human umbilical vein endothelial cells [38]. While these methods are promising, extensive characterization is still required to assess tissue health that recapitulates the in vivo human brain.

Generation and maintenance of cerebral organoids is largely unstandardized, and the resulting tissue has high variability across organoids and batches [39,40]. This limits the reproducibility of organoid studies, and thus is a barrier for the development of organoid-based drug-screening platforms. Currently, 3D organoids form spontaneously; thus, having a standardized scaffold available to which the organoid can conform will be beneficial for reducing the heterogeneity seen within batches. In contrast, having too much homogeneity may limit how well the tissue can recapitulate the human brain’s complexity.

## 6. Future Directions and Conclusions

In general, human neurodevelopment is a vastly complex process, and despite the enormous advancements in generating human-derived tissue in vitro, there is much more investigation needed to start incorporating organoids in the drug-screening pipeline. It is clear that organoids have the potential to advance personalized medicine, for neurological conditions such as drug-resistant epilepsy. It is necessary to better characterize the various neurotransmitter systems evident in the cerebral organoids. There are certain milestones, such as the “GABAergic switch”, that are integral to neurodevelopment, and thus would be imperative to demonstrate in tissue that is meant to model the developing brain. Such studies may include immunohistochemistry, PCR, RNA-Seq, and protein analyses of different receptors and their localizations at various timepoints. For example, in the previously discussed study [22], there is an eloquent characterization of RNA sequencing results over 6 months, and comparisons to human neonates. Similar work, complemented with functional analysis, is needed, with emphasis on the distribution of different neurotransmitter systems and how they relate to those observed in fetal development. Another future direction would be to optimize the growing conditions for cerebral organoids that are suitable for long-term culture, such as including vasculature, growing the tissue on microfluidic chip platforms, or slice culturing methods [41,42].

## Figures and Tables

**Table 1:** Percent difference in baseline to OGD + bumetanide spectral power.

Frequency	7-Month Average Baseline-OGD + Bumetanide PSD Percent Difference	4-Month Average Baseline-OGD + Bumetanide PSD Percent Difference	*p*-Value
Delta (1–3.9 Hz)	433.6	1103.6	0.26
Theta (4–7.9 Hz)	424.3	1652.0	0.25
Alpha (8–12.9 Hz)	393.4	1056.5	0.23
Beta (13–29.9 Hz)	321.0	877.2	0.21
Gamma (30–79.9 Hz)	159.6	479.1	0.14
High-Frequency Oscillations (80–119.9 Hz)	62.8	231.6	0.09
Very High-Frequency Oscillations (120–999.9 Hz)	6.4	30.2	0.04

## Data Availability

Data are available upon request from authors.

## Supplementary Materials

The following supporting information can be downloaded at: https://www.mdpi.com/article/10.3390/cells12151949/s1, Figure S1: Secondary antibody only stain; Figure S2: Sample OGD trace; Table S1: List of genes for qPCR, their forward and reverse primers, and associated references; Table S2: List of antibodies for immunofluorescence. References [43,44,45,46] are cited in the supplementary materials.
