# Supplementary material for: Treating Hyperexcitability in Human Cerebral Organoids Resulting from Oxygen-Glucose Deprivation
Source: Cells. 2023 Jul 27;12(15):1949. doi: 10.3390/cells12151949 (PMC10416870; doi:10.3390/cells12151949)
Supplement: Supplementary file 1 [file cells-12-01949-s001.zip › cells-2430582-supplementary.pdf]

Supplementary Material

1 Supplementary information for "Treating hyperexcitability in human cerebral organoids resulting from oxygen-glucose deprivation"

Santos et al.

2 **Figure S1:** Secondary antibody only stain

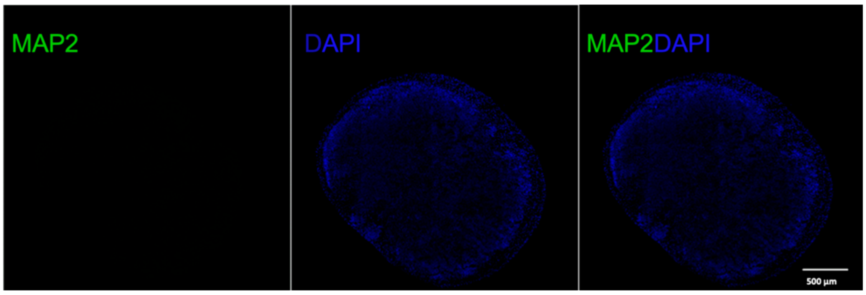

Supplementary Figure 1: Individual antibodies stained to confirm no non-specific binding in 7-month H9 cerebral organoids at 63X magnification oil immersion Zeiss LSM 880 Super resolution confocal microscope from University of Toronto Microscope Imaging Laboratory.

Figure S2: Sample OGD trace

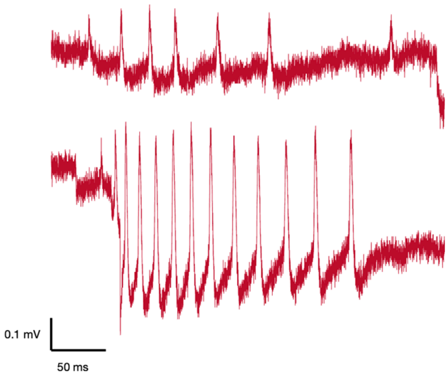

Supplementary Figure 2: Raw local field potential trace that demonstrate spiking events during OGD treatment.

3 Supplementary Tables

3.1 **Table S1:** List of antibodies for immunofluorescence

Antigen	Host	Dilution	Source	Identifier
MAP2	Chicken	1:1000	Abcam	ab5392
GFAP	Chicken	1:250	Abcam	ab4674

3.2 **Table S2:** List of genes for qPCR, their forward and reverse primers and associated references.

**Gene**	**Forward**	**Reverse**	**Reference**
CYC	CATACGGGTCCTGGCATCTTG	GCCATTCCTGGACCCAAAGC	NA
KCC2	ACATCTTTGGCGTCATCCTC	CAGGCACAACACCATTCGTT	43
NKCC1	CCGATTTTCGAGAGGAAGAG	TGCAATTCCTACGTAAACCAA	43
GAD67	CCTGGAACTGGCTGAATACC	CCCTGAGGCTTTGTGGAATA	44
GABRA1	GTCACCAGTTTCGGACCCG	AACCGGAGGACTGTCATAGGT	45
GABRA2	GTTCAAGCTGAATGCCCAAT	ACCTAGAGCCATCAGGAGCA	45
SOX2	CGAGGGAAATGGGAGGGGTGC	TGCAGCTGTCATTTGCTGTGGGT	46
MAP2	AGGCTGTAGCAGTCCTGAAAGG	CTTCCTCCACTGTGACAGTCTG	46
S100b	GAAGAAATCCGAACTGAAGGAGC	TCCTGGAAGTCACATTCGCCGT	4
